# Protein Nanoparticles Made of Recombinant Viral Antigens: A Promising Biomaterial for Oral Delivery of Fish Prophylactics

**DOI:** 10.3389/fimmu.2018.01652

**Published:** 2018-07-18

**Authors:** Rosemary Thwaite, Jie Ji, Débora Torrealba, Julio Coll, Manel Sabés, Antonio Villaverde, Nerea Roher

**Affiliations:** ^1^Department of Cellular Biology, Physiology and Immunology, Institute of Biotechnology and Biomedicine (IBB), Universitat Autònoma de Barcelona, Barcelona, Spain; ^2^Departamento de Biotecnología, Instituto Nacional Investigaciones y Tecnologías Agrarias y Alimentarias (INIA), Madrid, Spain; ^3^Biophysics Unit, Department of Biochemistry and Molecular Biology, Universitat Autònoma de Barcelona and ALBA Synchrotron, Barcelona, Spain; ^4^Institut de Biotecnologia i de Biomedicina, Universitat Autònoma de Barcelona, Barcelona, Spain; ^5^Departament de Genètica i de Microbiologia, Universitat Autònoma de Barcelona, Barcelona, Spain; ^6^Centro de Investigación Biomédica en Red (CIBER) de Bioingeniería, Biomateriales y Nanomedicina, Barcelona, Spain

**Keywords:** viral antigens, protein nanoparticles, fish, bacterial inclusion bodies, oral vaccines

## Abstract

In the search for an eminently practical strategy to develop immunostimulants and vaccines for farmed fish, we have devised recombinant viral antigens presented as “nanopellets” (NPs). These are inclusion bodies of fish viral antigenic proteins produced in *Escherichia coli*. Soluble recombinant proteins are too labile to endure the *in vivo* environment and maintain full functionality, and therefore require encapsulation strategies. Yet when they are produced as nanostructures, they can withstand the wide range of gastrointestinal pH found in fish, high temperatures, and lyophilization. Moreover, these nanomaterials are biologically active, non-toxic to fish, cost-effective regarding production and suitable for oral administration. Here, we present three versions of NPs formed by antigenic proteins from relevant viruses affecting farmed fish: the viral nervous necrosis virus coat protein, infectious pancreatic necrosis virus viral protein 2, and a viral haemorrhagic septicemia virus G glycoprotein fragment. We demonstrate that the nanoparticles are taken up *in vitro* by zebrafish ZFL cells and *in vivo* by intubating zebrafish as a proof of concept for oral delivery. Encouragingly, analysis of gene expression suggests these NPs evoke an antiviral innate immune response in ZFL cells and in rainbow trout head kidney macrophages. They are therefore a promising platform for immunostimulants and may be candidates for vaccines should protection be demonstrated.

## Introduction

Viral diseases are a major concern in the aquaculture industry. Vaccine strategies need to optimize efficacy, while taking into account production and administration costs, environmental risks, and compliance with legislation. The traditional approach is based on the use of inactivated or attenuated viral vaccines, which are commercially available for certain viral diseases that cause high mortality (1). Such vaccines induce a strong immune response when combined with oil adjuvants (2). However, not all fish viruses are readily culturable in cells, for example, lymphocystis disease virus (3), and the process is expensive, with administration *via* injection, or immersion for juveniles. Another consideration is the risk of possible reversion to virulence and environmental spread. New strategies are thus being sought. Among them, recombinant DNA vaccines have achieved promising results against certain viruses (4, 5) but raise safety issues regarding genetically modified organisms (6). In fact, only one DNA vaccine, Clynav^®^ (Elanco) against salmonid alphavirus subtype 3, has been recently licensed in Europe. Like other DNA vaccines, it is administered by labor intensive intramuscular injection. Injection is costly and difficult to perform on juveniles, as well as causing stress and injury to fish. An alternative vaccine approach is the use of recombinant protein viral antigens. These subunit vaccines can be produced in bulk, but have been variable in efficacy (1). One promising format, virus-like particles (VLPs), uses self-assembling viral capsid proteins produced in yeast, bacteria, or cell culture, drawing on advances in human and animal vaccinology (7, 8). The main advantage of subunit vaccines is they are safe. There is no risk of DNA integration into the host, reversion, or invasion. The main drawback is the stability and half-life of recombinant proteins *in vivo*. Oral delivery would be the most practical, least stressful delivery method; however, immunorelevant epitopes need to be protected against gastrointestinal pH, which is particularly low in carnivorous fish [see Figure 1 in Ref. (9)], as well as digestive enzymes within the tract. Thus different encapsulation techniques such as alginate and chitosan are being tested, aiming to protect the recombinant protein antigens from rapid degradation when inside the animal (10).

**Figure 1:**
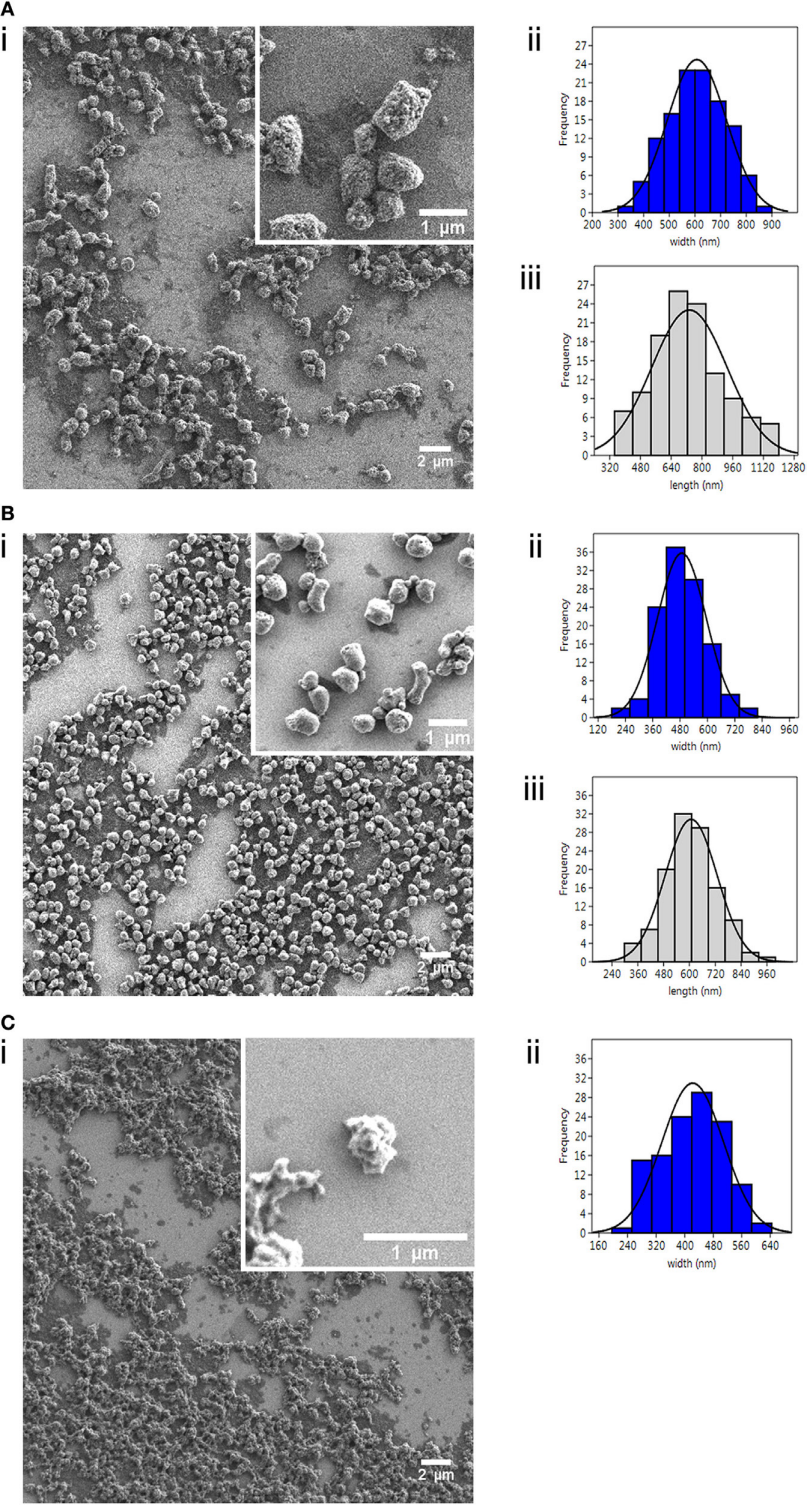
Characterization of nanostructured viral antigenic proteins. Field Emission Scanning Electron Microscopy images (i) of the three nanopellets (NPs): **(A)** IPNV-VP2^NP^, **(B)** VHSV-G-frg16^NP^, and **(C)** VNNV-C^NP^; with corresponding size distribution histograms (*n* = 120) for (ii) width (nm) and (iii) length (nm). Note there is no histogram (iii) for **(C)**, as these NPs were amorphic in length.

Here, we present a novel approach to finfish prophylactic design. To enhance the stability of antigenic proteins while maintaining functionality, we have nanostructured viral protein antigens as bacterial inclusion bodies (IBs). IBs are highly stable, tuneable, nanoscale protein particles which can penetrate cells, while retaining significant biological activity, as demonstrated by rescue studies (11). They can be designed to bear the antigenic protein/epitopes of interest and provide a slow release of functional protein over time (12). The attractiveness of IBs as a fish prophylactic is manifold. Their stability at gastrointestinal pH (13) would allow administrating the antigen orally through the feed, avoiding the necessity for vaccine encapsulation and the cost and stress to fish associated with injection. Production in *Escherichia coli* is achieved in bulk with a simple enzymatic and mechanical purification procedure which minimizes costs (14). This straightforward process implies that the IBs carry over fragments of bacterial lipopolysaccharide, peptidoglycans, and nucleic acids as impurities, but which are known adjuvants and immunomodulators of fish (15). The IB vehicle, a carrier and viral antigen as one biomaterial, should elicit both an innate and adaptive immune response against the target virus in fish. Finally, IBs’ stability under lyophilizing conditions and over a range of temperatures (13) indicates their potential as a practical farm product with a lasting shelf life, avoiding the cold chain.

We have already demonstrated the potential of IBs as an immunostimulant for fish, by nanostructuring recombinant cytokines TNF-α and CCL4 and testing them in a bacterial infection model in zebrafish (13). In addition, uptake of the TNF-α IB by intestinal cells was demonstrated *in vivo* in rainbow trout *via* oral intubation (13). This paved the way for work focusing on producing viral antigens as IBs, to explore this approach for immunostimulus, and ultimately as a viral vaccination strategy.

This paper is a proof-of-concept study concerning the production, uptake *in vitro* and *in vivo* and innate immunogenic potential of fish viral antigens configured as recombinant IBs. Given our final aim is their use in fish food, we have coined the term “nanopellets” (NPs) to refer to these novel nanostructured antigens. We chose three target proteins of known antigenicity from significant viruses affecting farmed finfish, reviewed in Ref. (6). They are the viral capsid protein 2 (VP2) from infectious pancreatic necrosis virus (IPNV) an *Aquabirnavirus* causing high mortality in young salmonids, the glycoprotein G of viral haemorrhagic septicemia virus (VHSV), a *Novirhabdovirus* which is a current OIE listed fish viral disease (http://www.oie.int/en/animal-health-in-the-world/oie-listed-diseases-2018/) primarily affecting farmed trout and turbot, and the C coat protein of viral nervous necrosis virus (VNNV), a *Betanodavirus* affecting sea bass, sea bream, flounder, and sole, among many other fish (16). We show the NPs can be produced by cost-effective, reproducible methods and can be taken up in ZFL (zebrafish liver cell line) and *in vivo* by zebrafish (*Danio rerio*) when orally administrated. Moreover, we show the viral antigen NPs can evoke an immune response *in vitro*, upregulating gene markers of the innate viral immune response in ZFL and rainbow trout (*Oncorhynchus mykiss*) head kidney macrophage cell cultures.

## Materials and Methods

### Design, Production, and Characterization of Nanostructured Viral Antigenic Proteins

#### Viral Strains and Plasmids

For the three target viruses, sequences for the antigenic proteins of interest were: VNNV coat protein gene from the Iberian betanodavirus isolate (strain SpSs-IAusc160.03), NCBI GenBank, accession no: NC_024493.1 which is a reassortant RGNNV/SJNNV strain (17); IPNV capsid protein 2 from the IPNV (strain Sp 31-75), Uniprot KB Q703G9 Chain (PRO_0000227873) position 1–442; VHSV glycoprotein G from the viral hemorrhagic septicemia virus (strain 07-71), Uniprot KB P27662. Clones were designed using the ORF and pET22b in a strategy removing the periplasmic location signal and including a C terminal polyHistag. Clones were codon optimized for expression in *E. coli*, synthesized by GeneArt (Thermo Fisher Scientific) and subcloned into pET22b. Recombinant plasmids were transformed into *E. coli* BL21(DE3) (Novagen). Upon protein production (see Production of NPs, Purification, Quantification, and Fluorescent Labeling) the VHVS-G protein showed hallmarks of being toxic for *E. coli*, with slow host growth and scant protein yield post production (data not shown). This clone was substituted by VHSV-G-frg16 cloned into pRSETa, which covers the C-terminal half (amino acid residues 252–450) of the VHSV (07-71) G protein sequence (NCBI Genbank X59148) to the 3′end, with the Cys residues mutated to Ser to facilitate expression in *E. coli*. The sequence includes a putative integrin receptor RGD-binding site and two regions which induce *Mx* gene expression (18, 19). Furthermore, frg16 is able to bind specific anti-VHSV rainbow trout antibodies in fish surviving VHSV infection (20). Apart from the viral antigen constructs, a construct with the red fluorescent protein (RFP), iRFPHis cloned in pET22b (Genscript), was also transformed into *E. coli* BL21(DE3) to be used as a non-immune-relevant control protein.

#### Production of NPs, Purification, Quantification, and Fluorescent Labeling

Production of nanostructured viral and control proteins from the clones transformed into *E. coli* followed the method described in Ref. (13). Briefly, *E. coli* was cultured in LB with ampicillin (Sigma-Aldrich) at 100 µg/ml, and recombinant protein expression was induced with 1 mM IPTG (Panreac) when OD_550 nm_ reached 0.5–0.8. After a further 3 h growth at 37°C, IBs were isolated *via* a straightforward enzymatic and mechanical disruption of the cells according to Ref. (13). Finally, the nanostructured proteins were subject to sterility tests without antibiotic on LB-agar overnight and in DMEM culture medium (Gibco) at 37°C for 3 days. Pellets of purified NPs, named IPNV-VP2^NP^, VHSV-G-frg16^NP^, and VNNV-C^NP^, were stored at −80°C until use. Protein was quantified by western blot using an anti-His-tag antibody (Genscript A00186-100), and the protein concentration was calculated from a standard curve using recombinant protein and Quantity One software (Bio-Rad). Quantification was further tuned *via* spectrometry by comparing 100 µg/ml dilutions of the different NPs at 320 nm and using the correction factor determined to adjust the quantification accordingly. For experiments to visualize the nanoparticles by flow cytometry or confocal microscopy, NPs were conjugated with Atto-488 NHS ester (Sigma-Aldrich) according to the manufacturer’s instructions. Labeling efficiency was determined on a fluorometer (Jasco FP8200). Equal volumes of nanoparticles at 100 µg/ml were treated with 6 M guanidinium chloride (Sigma-Aldrich) to denature overnight (O/N) at room temperature (RT) in the dark and the fluorescence intensity was read the next morning (see Figure S1 in Supplementary Material).

#### Characterization of Viral Recombinant Protein NPs

We used Field Emission Scanning Electron Microscopy (FESEM, Zeiss Merlin) to determine the external morphology and physical dimensions of the NPs. Samples were prepared by resuspending NPs at 100 µg/ml in distilled water, pipetting 20 µl onto silicon chips, and air drying O/N. Images were analyzed using Fiji open source image processing package (21), measuring the dimensions of a minimum of 120 particles for each construct. Size distribution histograms were generated using Past3 software (v3.18, University of Oslo).

### *In Vitro* Assays

#### Cell Cultures

Zebrafish ZFL cells (CRL-2643, ATCC) were cultured according to Ref. (22) at 28°C and 5% CO_2_ in DMEM + GlutaMAX (Gibco), 10% heat-inactivated fetal bovine serum (FBS) (Gibco), 0.01 mg/ml insulin (Sigma-Aldrich), 50 ng/ml epidermal growth factor (Sigma-Aldrich), 2% (v/v) antibiotic/antimycotic (Gibco), and 0.5% (v/v) trout serum which had been filtered (0.20-µm filter Corning) and heat inactivated for 30 min at 45°C, before storing at −20°C. Rainbow trout head kidney macrophages (RT-HKM) were isolated from *O. mykiss* (109 ± 18 g body weight) following previously described procedures (23). Primary adherent cultures were established in DMEM + GlutaMAX, 10% heat-inactivated FBS and 100 µg/ml Primocin (Invitrogen) at 16°C and 5% CO_2_. Experiments for NP uptake and gene expression were performed on day 5 when the macrophages were fully differentiated.

#### Uptake of Nanostructured Viral Antigens by ZFL

To test cellular uptake, fluorescently labeled NPs were added to ZFL cultures at 70% confluence after 2–3 h incubation in minimal media (0–0.5% FBS) at the doses and times indicated below. For dose–response assays, VNNV-C^NP^ and IPNV-VP2^NP^ were added at 5, 10, and 20 µg/ml; and VHSV-G-frg16^NP^ at 1, 5, 10, and 20 µg/ml. Cultures were then incubated O/N (12–14 h). In time course experiments, NPs were added at 10 µg/ml for VNNV-C^NP^ and IPNV-VP2^NP^; and at 5 µg/ml for VHSV-G-frg16^NP^ and cultures were simultaneously incubated for 6–48 h before harvesting. Both dose–response and time course experiments were performed in duplicate. Post treatment, cells were washed in PBS and incubated at 28°C with 1 mg/ml Trypsin (Gibco) for 15 min. This strong trypsinization step aimed to remove NPs attached to the cell surface (24). Then, two volumes of complete medium were added, and cells were retrieved by centrifugation at 300 × *g* for 5 min. Pellets were resuspended in PBS for flow cytometry (FACSCalibur BD), and 10,000 events were counted. Data were analyzed using Flowing Software 2.5.1 (University of Turku, Finland) and plotted with Prism 6.01 (GraphPad). A one-way ANOVA was performed with Dunnett’s multiple comparisons test, comparing treatment and control means. To confirm the fluorescent NPs were inside the cells, we performed confocal microscopy (Zeiss LSM 700). ZFL cells were seeded on Nunclon Δ Surface individual well plates (Nunc). The next day cells at approximately 60% confluence were placed in minimal media. NPs were added 2–3 h later as follows: VNNV-C^NP^ and IPNV-VP2^NP^ at 20 µg/ml and VHSV-G-frg16^NP^ at 10 µg/ml. Cells were incubated for 14 h at 28°C. Medium was replaced with minimal media in which the cells were stained with DAPI (nuclei) and Cell mask Deep Red (membrane) (Life Technologies). Images were analyzed using Imaris software v8.2.1 (Bitplane).

#### NP Cytotoxicity Studies in ZFL

Cytotoxic and cytostatic effects of NPs on ZFL were checked using an MTT assay. After 2.5 h on minimal media, cultures were stimulated with NPs at 10, 20, and 50 µg/ml and incubated for 14 h at 28°C. Cells were washed in PBS and MTT substrate (Sigma-Aldrich) was added to 10% total volume. Controls were cells with no NPs, cells with no NPs but treated with 1% Triton (Sigma-Aldrich) before adding MTT, and cells with no NPs and no MTT. Cells were further incubated at 28°C for 6 h. The solution was removed, cells were solubilized in DMSO and the lysate read on Victor 3 (PerkinElmer) at 550 nm. The experiment was repeated twice. Data were normalized using Prism 6.01 (Graph Pad) such that the control readings were set at 100% and the Triton treatment readings were 0% viability, being equivalent to cells without MTT. A one-way ANOVA was performed with Dunnett’s multiple comparisons test, comparing treatment and control means.

#### Gene Expression Analysis in ZFL and RT-HKM Treated With NPs

ZFL cells at 60% confluence were cultured in minimal media (0–0.5% FBS) for 2–3 h and then stimulated for 14 h with NPs at the following concentrations in triplicate: VNNV-C^NP^ and IPNV-VP2^NP^ at 10 µg/ml, VHSV-G-frg16^NP^ at 5 µg/ml. Controls were poly(I:C) 25 µg/ml (Sigma-Aldrich) as a viral dsRNA mimic and iRFP^NP^ at 10 µg/ml as an immunogenically irrelevant protein, as well as control cells with no stimulus. Total RNA was extracted using TriReagent (Sigma-Aldrich) following the manufacturer’s instructions. RNA was quantified using the nanodrop ND-1000 (Thermo Fisher Scientific) and integrity was checked on the Agilent 2100 Bioanalyzer using the RNA 6000 Nano Lab-Chip kit (Agilent Technologies). The experiment was repeated, and four complete sets of high quality RNA from two independent experiments were selected for cDNA synthesis using 1 µg of total RNA and iScript cDNA synthesis kit (Bio-Rad). Quantitative real-time PCR (qPCR) was performed at 60°C annealing temperature using iTaq Universal SYBR Green Supermix (Bio-Rad) with 250 nM of primers and 2.5 µl of cDNA previously diluted to 1:25 for the target and 1:500 for the reference gene, elongation factor 1 alpha (*ef1-*α) (25). Primers were designed for six zebrafish gene markers of the innate immune response to viral infection (*mx, viperin (vig1), gig2, irf7, stat1b*, and *ccl4*) using NCBI Primer BLAST, and revised using Oligoanalyzer 3.1 (Integrated DNA Technologies). The primer sequences and accession numbers are listed in Table S1 in Supplementary Material. All the samples (*N* = 4 per treatment) were run in triplicate, and data were analyzed for individual replicates using the Livak method (26). Statistical analysis used a one-way unpaired *t*-test to compare each gene’s mean fold change in expression with control using Welch’s correction for unequal variances (Prism 6.01, GraphPad).

A further gene expression experiment was carried out in RT-HKM primary cultures using the two NPs made with antigenic proteins from virus affecting salmonids, IPNV and VHSV. The macrophage cultures were prepared as described in Section “Cell Cultures.” On day 5, cultures from three trout at approximately 70% confluence were placed in serum-free media for 2 h at 16°C. Cultures were stimulated for 15 h as follows: IPNV-VP2^NP^ and VHSV-G-frg16^NP^ at 10 µg/ml; and controls: poly(I:C) at 10 µg/ml and iRFP^NP^ at 10 µg/ml, as well as cells with no stimulus. The experiment was repeated twice. Total RNA was extracted and quantified as described above for ZFL. From the two independent experiments, four sets of high quality RNA were selected for cDNA synthesis and qPCR as described above. The trout primer sequences were obtained from published papers or were designed with NCBI primer BLAST, selecting genes which were reported to be upregulated in VHSV infection of *O. mykiss* (27). The reference gene used was *ef1-*α (28) with cDNA diluted to 1:500. The dilution factor for the other genes tested was 1:50 (*vig1, mx*, and *ccl4*) or 1:25 (*ifit5* and *mda5*). The primer sequences and accession numbers are listed in Table S1 in Supplementary Material. Data analysis was performed as described above.

### *In Vivo* Assays

#### Animals

Adult wild-type zebrafish (*D. rerio*) and rainbow trout (*O. mykiss*) fish were maintained at 27 ± 1 and 17 ± 1°C, respectively, in a 12 h light/dark cycle, fed twice daily with a commercial diet at 2% ratio. All animal experiments were performed in accordance with the ethics statement at the end of the manuscript.

#### Uptake of NPs by Zebrafish *via* Oral Intubation

To test *in vivo* uptake of NPs, the fluorescently labeled nanoparticles were intubated in zebrafish adults for the indicated times and doses, mimicking an oral vaccine administration route. Zebrafish adults (mean weight 0.9 ± 0.2 g) were acclimatized in tanks without feeding for 1.5 days prior to the experiment. Atto labeled NPs were intubated into the animals in a volume of 30 µl PBS using a gastight Hamilton syringe (Hamilton Company) with a thin silicon tube (0.30 mm inner diameter, Dow Corning) placed over the needle as a protective sheath to avoid injuring the animal. To guide oral insertion, a more rigid 10 µl filtered pipette tip end (NerbePlus) was cut and fixed over the tubing leaving the soft end exposed. Immediately prior to intubation, fish were anesthetized in 120–140 mg/l MS-222 (tricaine mesylate) (Sigma-Aldrich). Preliminary small scale runs at 3, 6, 24, and 48 h at 20 µg and 50 µg/fish indicated maximum uptake was achieved by 6 h and 20 μg/fish dose was sufficient. Then runs were performed with groups of *N* = 8 fish for each NP at 20 µg/fish in 30 µl PBS for 5 h. Controls were fish intubated with 30 µl PBS without NP. Post administration, fish were maintained in tanks until time of sacrifice using an overdose of MS-222. The intestine was dissected out from euthanized fish and washed in PBS. Next, it was incubated in 1 ml of collagenase solution: DMEM (Gibco) with 1% v/v antibiotic/antimycotic (Gibco) and collagenase Type IV (Gibco) 1.5 mg/ml at RT on a roundabout in the dark for 1 h. The intestine was passed through a 100-µm cell strainer (Falcon, Corning), washing with PBS and cells were retrieved by centrifugation at 400 × *g* for 10 min at 4°C. Cells were resuspended in PBS for flow cytometry (FACSCalibur BD), and 10,000 events were counted. Data were analyzed using Flowing Software 2.5.1 (University of Turku, Finland) and plotted with Prism 6.01 (GraphPad). A one-way unpaired *t*-test with Welch’s correction for unequal variances was performed to test equivalence of means between each experimental group and controls.

### Statistical Analysis

Analyses were performed with Prism 6.01 software (GraphPad), and Imaris 8.2.1 (Bitplane) for the confocal images and Past3 (v3.18, University of Oslo) for data obtained from FESEM. Data are shown as mean ± SD. Comparisons of means for each experimental group versus control were performed using a one-way unpaired *t*-test with Welch’s correction for unequal variances. For the *in vitro* uptake studies, in which we compared a series of conditions with the same NP, a one-way ANOVA was used, followed by Dunnett’s multiple comparisons test for each treatment versus control; *p* < 0.05 was considered statistically significant in all analyses.

## Results

### Characterization of Nanostructured Viral Antigenic Proteins

We successfully produced the three viral proteins in *E. coli* as bacterial IBs (i.e., NPs) (Figure S2 in Supplementary Material) with yields post purification as follows: IPNV-VP2^NP^ 104 mg/l, VHSV-G-frg16^NP^ 120 mg/l, and VNNV-C^NP^ 50 mg/l. The NPs had distinct morphologies and sizes as seen in the FESEM images (Figure 1). IPNV-VP2^NP^, the largest of the NPs, is generally barrel shaped and porous; VHSV-G-frg16^NP^ is rounder and smoother, while VNNV-C^NP^ has an irregular surface with small spherical protrusions. We have observed similar morphologies in other IBs produced in *E. coli* in the same strain BL21(DE3) and in M15(pREP4) (13). The size range is shown in Figure 1 (ii and iii) with average width and length being 607 ± 115 and 734 ± 195 nm for IPNV-VP2^NP^; 488 ± 107 and 608 ± 121 nm for VHSV-G-frg16^NP^, respectively, and 422 ± 87 nm for VNNV-C^NP^ mean width. The morphological features of the nanostructured control protein iRFP^NP^ have already been published (14).

### Uptake of Viral NPs by ZFL

**Figure 2:**
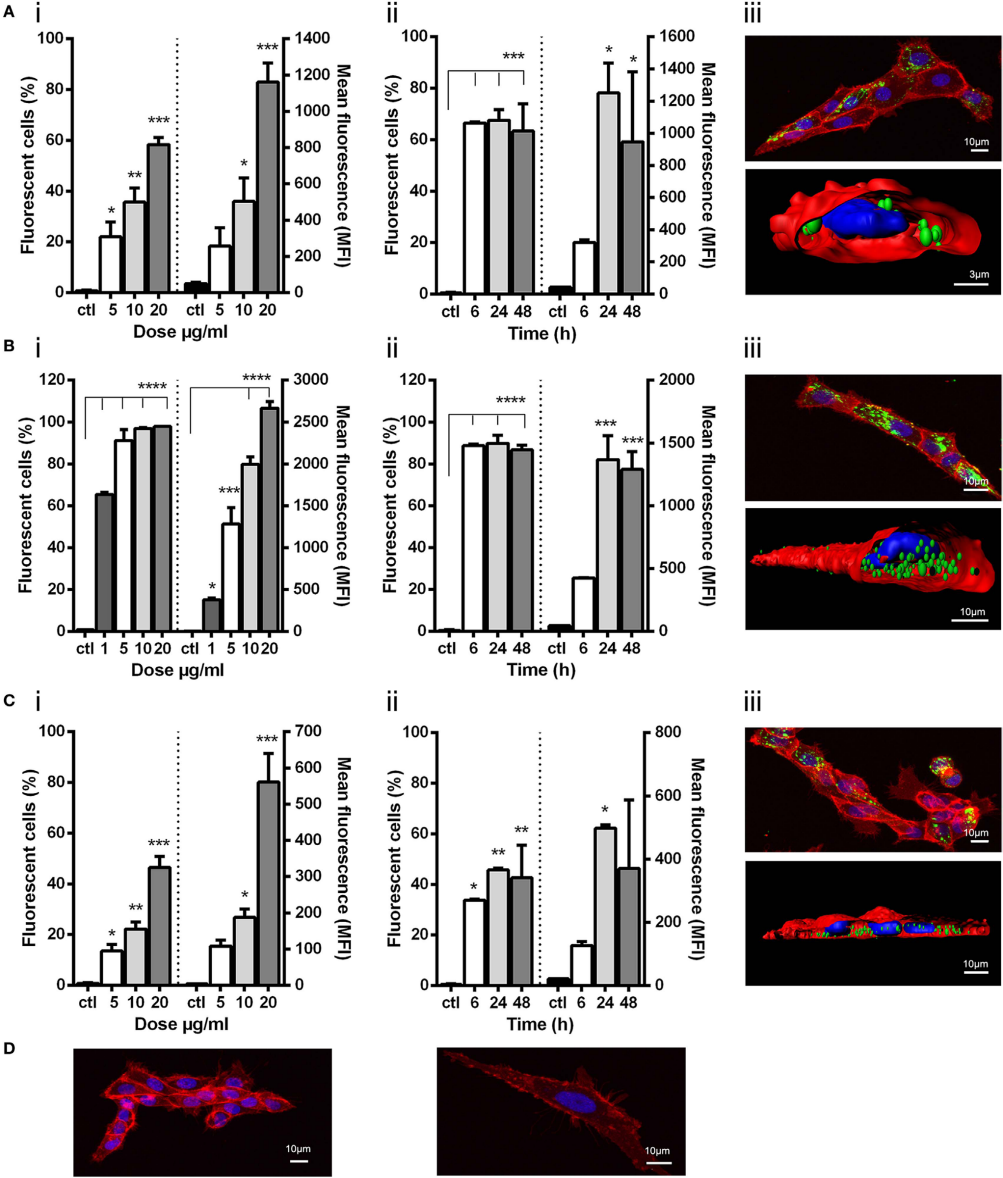
Uptake of viral nanopellets (NPs) by ZFL. Fluorescently labeled NPs **(A)** IPNV-VP2^NP^, **(B)** VHSV-G-frg16^NP^, and **(C)** VNNV-C^NP^ were added to ZFL. Control (ctl) was ZFL without NPs. (i) Dose–response. Cells incubated for 12 h with NPs **(A,C)** at 5–20 µg/ml, and **(B)** at 1–20 µg/ml in duplicate. (ii) Time course. NPs added to cells at 10 µg/ml **(A,C)**, and 5 µg/ml **(B)** in duplicate and incubated for 6–48 h. Differences between means were analyzed by a one-way ANOVA with Dunnett’s multiple comparisons test, treatments versus control. Significance levels *p < 0.05; **p < 0.01; ***p < 0.001; ****p < 0.0001. (iii) Confocal microscopy and digitalized image (z-stacks) of ZFL cells after 14 h incubation with 20 µg/ml **(A,C)** and 10 µg/ml **(B)**. NPs are green, cell membrane red, and nuclei blue. Control confocal image ZFL without NPs **(D)**.

All three NPs were taken up by ZFL cells. In dose–response experiments, uptake of VHSV-G-frg16^NP^ was found to be particularly efficient, achieving ~100% fluorescent cells at 10 µg/ml O/N [Figure 2B (i)]. Hence, an additional lower dose (1 µg/ml) for this NP was included in subsequent experimental runs. For IPNV-VP2^NP^ and VNNV-C^NP^, uptake increased progressively with dose, reaching a maximum of ~60 and 50% fluorescent cells, respectively [Figure 2A (i) and Figure 2C (i)]. In all cases, the mean fluorescence intensity (MFI) increased with dose, indicating susceptible cells were still able to take up more NP [Figures 2A–C (i) right *y* axis]. For time course experiments, a fixed dose was chosen that achieved less than the maximum uptake observed in the dose–response experiments. 10 µg/ml for IPNV-VP2^NP^ and VNNV-C^NP^, and 5 µg/ml for VHSV-G-frg16^NP^. In the time course experiments, IPNV-VP2^NP^ and VHSV-G-frg16^NP^ already reached the maximum percentage of fluorescent cells by 6 h [Figures 2A,B (ii)]. For VNNV-C^NP^ uptake was slower, as the maximum percentage of fluorescent cells for the time points measured was at 24 h [Figure 2C (ii)]. In all cases, by 48 h, the percentage of fluorescent cells had started to drop [Figures 2A–C (ii)], possibly indicating the NPs had begun to be metabolized. The MFI results for the time course are consistent with this. Susceptible cells continued taking up NPs for the first 24 h, then between 24 and 48 h the MFI dropped [Figures 2A–C (ii) right *y* axis].

The confocal microscopy images for IPNV-VP2^NP^ and VNNV-C^NP^ [Figures 2A,C (iii)] show that there are cells which have taken up a lot of NP, but others which have very few or no NPs. This is consistent with the cytometry results in which the maximum percentage of fluorescent cells which took up these particles O/N, at the same dose as the confocal experiments (20 µg/ml), were ~60 and 50%, respectively [Figures 2A,C (i)]. There are therefore some cells which do not up take IPNV-VP2^NP^ and VNNV-C^NP^ under these conditions. By contrast, all cells we observed in confocal microscopy had taken up VHSV-G-frg16^NP^ in large quantities. This concords with the O/N cytometry results at the same dose (10 µg/ml), which reached 100% fluorescent cells [Figure 2B (i)]. The digitalized z-stack images [Figure 2 (iii)] clearly show all three NPs have been internalized by the cells. For VHSV-G-frg16^NP^, some particles are also visibly embedded in the membrane and numerous NPs are inside the cell [Figure 2B (iii)]. The Imaris imaging software allows estimating the number of nanoparticles per cell. In a small sample, the NPs/ZFL cell were as follows (mean and SD): IPNV-VP2^NP^, 50 ± 19 NPs/cell and 67% of cells counted had NPs (*n* = 9); VNNV-C^NP^, 57 ± 31 NPs/cell and 65% of cells had NPs (*n* = 20); VHSV-G-frg16^NP^, 88 ± 45 NPs/cell and 100% of cells had NPs (*n* = 11).

Finally, the MTT assays in ZFL incubated with 10, 20, and 50 µg/ml of each NP for 14 h showed no significant difference in survival between control and any treatment group indicating that none of the NPs are cytotoxic (see Figure S3 in Supplementary Material). Moreover, in the intubation experiments in zebrafish up to 48 h (see Uptake of NPs by Zebrafish *via* Intubation) fish showed no signs of malaise. In fact, we have previously injected up to 300 µg/fish of nanostructured TNF-α and maintained the animals for 30 days with no signs of any deleterious effects (13).

### Gene Expression Analysis in ZFL Stimulated With NPs

**Figure 3:**
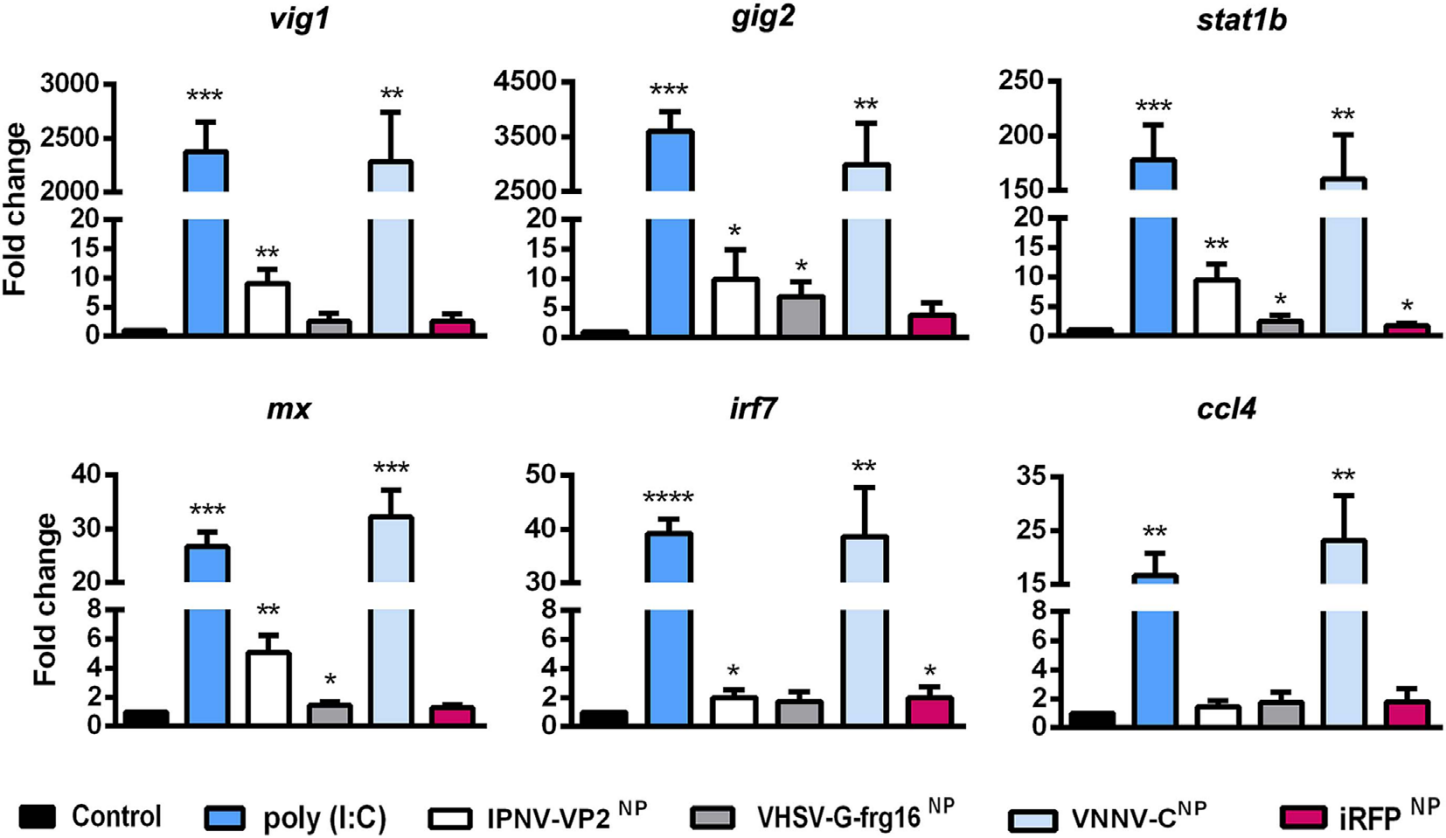
Gene expression analysis in ZFL stimulated with nanopellets (NPs). Cells were incubated for 14 h as follows: unstimulated control cells (black), poly(I:C) 25 µg/ml as a positive control (blue), IPNV-VP2^NP^ 10 µg/ml (white), VHSV-G-frg16^NP^ 5 µg/ml (gray), VNNV-C^NP^ 10 μg/ml (light blue), and iRFP^NP^ 10 µg/ml as an immunogenically irrelevant NP control (red). Samples are from two independent experiments. Data are mean ± SD (*n* = 4). Gene expression was determined by quantitative real-time PCR with three technical replicates. Differences between each treatment mean and control were analyzed by unpaired one-sided *t*-tests with Welch’s correction for unequal variances. Significance levels *p < 0.05; **p < 0.01; ***p < 0.001; ****p < 0.0001.

To see whether the NPs could elicit an innate immune response in line with that provoked by viral infection, ZFL were stimulated with the three viral NPs O/N at 10 µg/ml for IPNV-VP2^NP^ and VNNV-C^NP^ and 5 µg/ml for VHSV-G-frg16^NP^. We used half of the dose of VHSV-G-frg16^NP^ compared with the other NPs, given that uptake of this nanoparticle in ZFL had been greater than the others, even at this lower dose (see Uptake of NPs by Zebrafish *via* Oral Intubation and Figure 2B). We used poly(I:C) (25 µg/ml) as a viral dsRNA mimic, and iRFP^NP^ (10 µg/ml) as a control NP made with an immunogenically irrelevant protein. Gene expression of six gene markers of the innate immune response to viral infection was tested by qPCR (Figure 3). For all genes tested, there was a remarkable similarity in the response to poly(I:C) and VNNV-C^NP^, significantly different from the untreated control. For *vig1* and *gig2*, the upregulation was several thousand-fold for both treatments. For *stat1b*, the mean fold change (±SD) was 178 ± 32 for poly(I:C) stimulated cells and 160 ± 41 for ZFL stimulated with VNNV-C^NP^. *Mx* and *irf7* were upregulated between 27 ± 3- and 39 ± 3-fold by both treatments, while *ccl4* was upregulated 17 ± 4- and 23 ± 8-fold by poly(I:C) and VNNV-C^NP^, respectively. For the other two viral NPs, the fold change in gene expression was positive but much lower. IPNV-VP2^NP^ elicited a statistically significant upregulation for all genes except *ccl4*, ranging from 9 ± 2.4-fold for *vig1* to 2 ± 0.5-fold for *irf7*. VHSV-G-frg16^NP^ only elicited a significant upregulation for three of the genes tested: *gig2*, 7 ± 2.5-fold; *stat1b*, 2.5 ± 1.0-fold, and *mx* 1.5 ± 0.2-fold. iRFP^NP^ was significantly, though slightly upregulated for two of the genes tested: 2 ± 0.8- and 1.7 ± 0.4-fold for *irf7* and *stat1b*, respectively.

### Gene Expression Analysis in RT-HKM Stimulated With Salmonid Viral NPs

**Figure 4:**
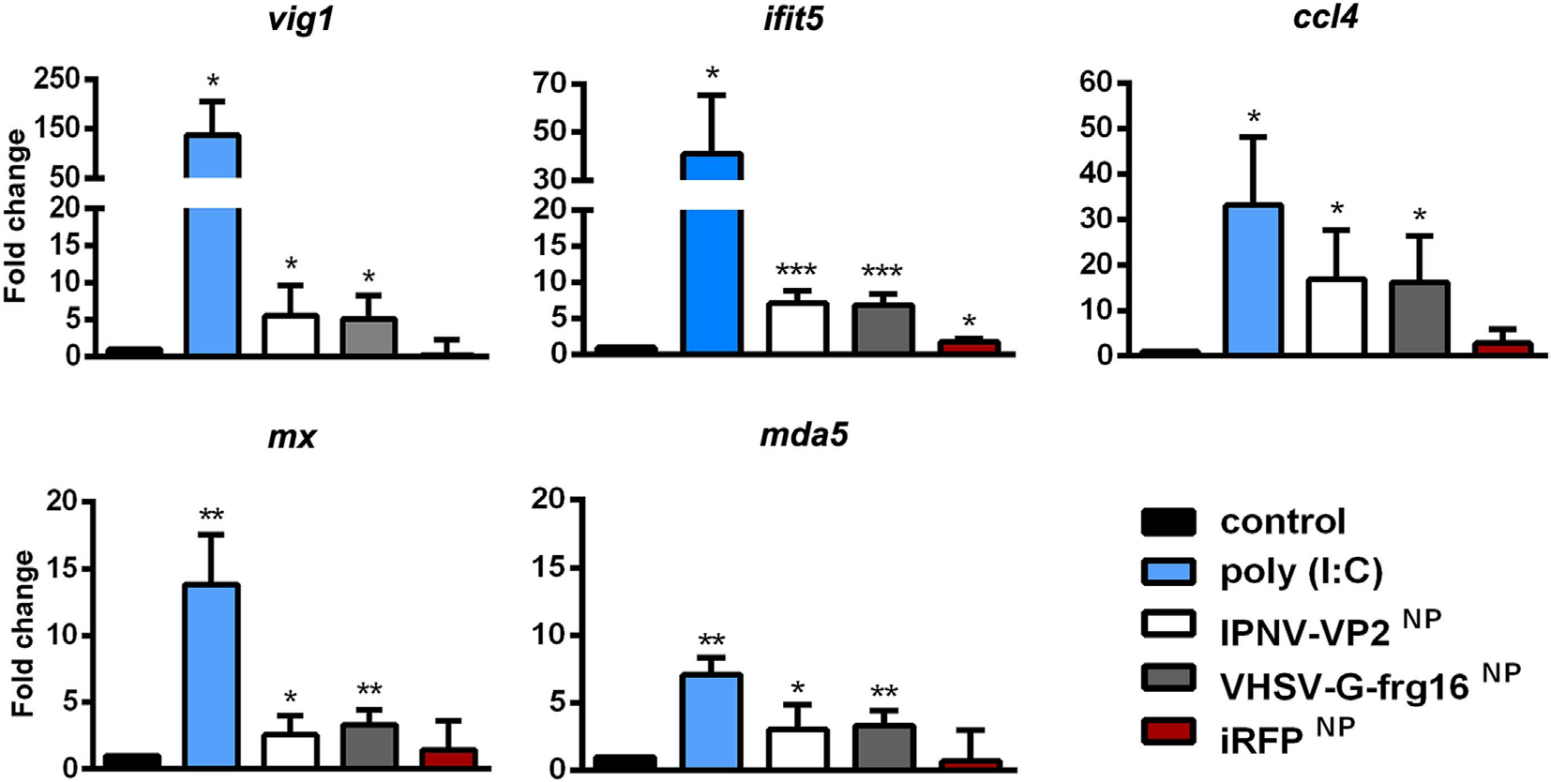
Gene expression analysis in RT-HKM stimulated with salmonid viral nanopellets (NPs). Cells were incubated for 15 h as follows: unstimulated control cells (black), poly(I:C) 10 µg/ml as a positive control (blue), IPNV-VP2^NP^ 10 µg/ml (white), VHSV-G-frg16^NP^ 10 µg/ml (gray), and iRFP^NP^ 10 µg/ml as an immunogenically irrelevant NP control (red). Samples are from two independent experiments. Data are mean ± SD (*n* = 4). Gene expression was determined by quantitative real-time PCR with three technical replicates. Differences between each treatment mean and control were analyzed by unpaired one-sided *t*-tests with Welch’s correction for unequal variances. Significance levels *p < 0.05; **p < 0.01; ***p < 0.001.

As the innate immune response to VHSV-G-frg16^NP^ had been weak in ZFL except for *gig2*, we decided to test the NP-based stimulus in RT-HKM primary cultures. Using macrophages from trout, a natural host for VHSV and IPNV would provide more pertinent *in vitro* data for the two NPs formed by salmonid viral antigenic proteins. We therefore incubated RT-HKM with IPNV-VP2^NP^ and VHSV-G-frg16^NP^ as well as poly(I:C) and iRFP^NP^ controls all at 10 µg/ml. Genes tested included *vig1, mx*, and *ifit5* which are relevant markers of VHSV infection (27), as well as *mda5* and *ccl4*. For all genes tested, both IPNV-VP2^NP^ and VHSV-G-frg16^NP^ evoked upregulation, significantly different from the untreated control (Figure 4) as follows: *vig1* 5.6 ± 4.1 and 5.1 ± 3.2-fold for IPNV-VP2^NP^ and VHSV-G-frg16^NP^, respectively; continuing in that order *ifit5* 7.1 ± 1.7 and 6.9 ± 1.6; *ccl4* 16.9 ± 10.8 and 16.2 ± 10.2; *mx* 2.6 ± 1.4 and 3.3 ± 1.1; *mda5* 3.0 ± 1.8 and 3.3 ± 1.2. For all genes tested, the poly(I:C) positive control elicited higher upregulation than the NPs, but the difference was not as great as seen in ZFL. Note in this case, the poly(I:C) dose used was the same (10 µg/ml) as for the NPs whereas in ZFL we used 25 µg/ml (29). The most similar response to stimulus with the NPs was seen in *mda5* which was upregulated 7.1 ± 1.3 with poly(I:C) treatment. iRFP^NP^ treatment only significantly upregulated 1 gene very weakly, *ifit5* 1.8 ± 0.4-fold.

### Uptake of NPs by Zebrafish *via* Intubation

**Figure 5:**
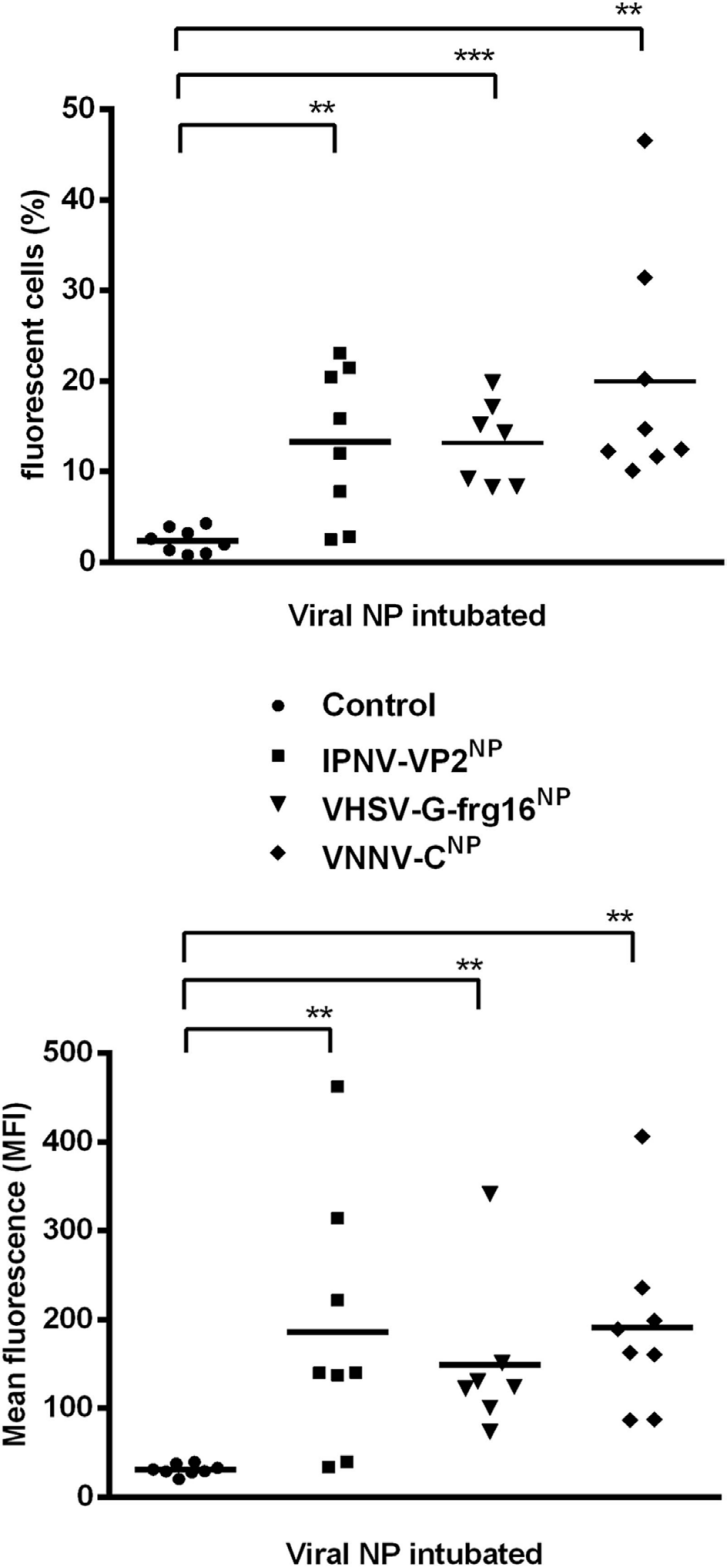
Uptake of nanopellets (NPs) by zebrafish *via* intubation. Adult zebrafish were intubated with 20 μg/fish of each fluorescently labeled NP in 30 µl PBS for 5 h (*n* = 8), then intestine cells were sampled for cytometry. ● Control fish: 30 µl PBS intubated without NP. Each point represents data from one fish intubated with ■ IPNV-VP2^NP^, ▾ VHSV-G-frg16^NP^, or ♦ VNNV-C^NP^. Horizontal bars are the means. Differences between the mean of each treatment group and control were analyzed by an unpaired one-sided *t*-test with Welch’s correction for unequal variances. Significance levels *p < 0.05; **p < 0.01; ***p < 0.001.

In preliminary *in vivo* experiments, adult zebrafish (*n* = 3) were intubated with the viral NPs at 20 and 50 μg/fish and sampled at 6, 24, and 48 h. By 24 h, the percentage of fluorescent cells had dropped by approximately 50% compared with 6 h and had dropped further by 48 h, indicating early uptake of the NPs *in vivo* (data not shown). Hence, the intubation experiments with larger numbers of fish, reported here (Figure 5), were done at a short time interval of 5 h. Adult zebrafish were able to take up the three viral NPs into gut cells when administered orally *via* intubation at 20 μg/fish. For IPNV-VP2^NP^, 75% of the fish intubated had taken up the NP after 5 h, while for VHSV-G-frg16^NP^ and VNNV-C^NP^, 100% of the fish intubated internalized the NPs (*n* = 8). The range and mean of the percentage of fluorescent cells (10,000 events) (Figure 5 upper graph) were: range 0–23%, mean 13% for IPNV-VP2^NP^, range 8–19%, mean 13% for VHSV-G-frg16^NP^, and range 10–47%, mean 20% for VNNV-C^NP^. The MFI results (Figure 5 lower graph) in general clustered around the average for each group, being 186, 151, and 191 for IPNV-VP2^NP^, VHSV-G-frg16^NP^, and VNNV-C^NP^, respectively. Note the fluorescence labeling efficiency with Atto-488 NHS was lower for VHSV-G-frg16^NP^ compared with the other two NPs (see Figure S1 in Supplementary Material). This explains the lower average MFI in intestine cells which had taken up VHSV-G-frg16^NP^.

## Discussion

The thrust of our work is to seek a safe and effective, but eminently practical solution for fish vaccination in the long term. To this purpose, we have successfully produced three viral antigenic proteins in *E. coli* as IBs. The production of these “nanopellets” followed a simple, fully scalable, batch culture procedure in *E. coli*, with isolation by mechanical and enzymatic methods. This is a more straightforward, less costly approach than that required to produce VLPs (8), or purified soluble recombinant proteins and avoids safety issues raised regarding DNA vaccines. Concerning biocompatibility, the NPs were not toxic to ZFL cells nor were there any signs of malaise in adult zebrafish intubated with up to 50 µg/fish for 48 h. In previous work, we have injected IBs at up to 300 µg/zebrafish and maintained the animals for 30 days with no signs of any deleterious effects (13). We therefore consider the recombinant protein NPs are innocuous to fish. Having successfully produced the NPs, we wished to address two critical questions at this stage: Could the NPs be taken up in fish? And, would an initial immune response be evoked, given the importance of the innate immune response in establishing an effective adaptive immune response to vaccination (30)?

As regards uptake, an advantage of IBs is that the amyloid scaffold can protect the functional protein from degradation while passing through the low pH of the gastrointestinal tract. We have already successfully tested other NPs resistance at pH 2.5 and uptake in intubated trout (13). The scaffold itself is resistant to proteinase K digestion but represents approximately 20% of the protein in the structure (31), leaving a considerable amount of functional protein to be released slowly within the organism. Here, we tested first, uptake *in vitro* in ZFL and then *in vivo* in zebrafish *via* intubation. In ZFL all three NPs were taken up O/N, achieving ≥ 50% of the cells sampled. VHSV-G-frg16^NP^ uptake was strikingly efficient even at 6 h, the earliest time point tested. The abundant uptake of VHSV-G-frg16^NP^ by ZFL was corroborated by the confocal microscopy results. For the two other NPs, uptake was also high in susceptible cells, but not all cells had internalized the particles. The VHSV-G-frg16^NP^ construct contains an arginine-glycine-aspartic acid (RGD) tripeptide integrin binding site (18, 19), not present in IPNV-VP2^NP^ nor VNNV-C^NP^. RGD-binding integrins are known receptors or coreceptors for certain viruses (32). In addition, in experiments on IB uptake in HeLa cells, an IB with the RGD site mutated to RGE was internalized significantly less than that with RGD (24). We thus hypothesized the RGD site in VHSV-G-frg16^NP^ may be facilitating IB uptake in ZFL.

The *in vivo* uptake results in zebrafish were also encouraging. The three NPs were able to be taken up by almost all fish tested *via* the intestine in a matter of hours. The zebrafish gut is composed of intestinal epithelial cells, goblet cells, smooth muscle cells [see Figure 1A in Ref. (33)], and immune cells also known as gut-associated lymphoid tissue (GALT). The fish GALT is less structured than the mammalian GALT. It contains two main populations of immune cells: the leukocytes in the lamina propria, which include various immune cells, such as granulocytes, macrophages, lymphocytes, and plasma cells; and intraepithelial lymphocytes, composed of T cells and some B cells located among epithelial cells. These immune cells together regulate gut immune responses. The GALT is particularly important because it is the main immune tissue involved in the uptake and processing of orally administrated antigens (10). We found an average of 13, 13, and 20% of cells had taken up IPNV-VP2^NP^, VHSV-G-frg16^NP^, and VNNV-C^NP^, respectively, 5 h after oral administration of a single dose. We do not know which specific cell type is taking up the viral NPs but in previous work we have shown that cytokine-made NPs can be found in the lamina propria (midgut) and in the villi apex where lymphoid cells are located (13).

The development of the zebrafish intubation method used should also be noted. We are able to successfully administer up to 30 µl, to fish of mean weight 0.9 ± 0.2 g simply and quickly, without injuring the animals. The fact that fish were able to take up the NPs *via* the oral route is crucial as a proof of concept for a strategy to evoke mucosal immune stimulus. Nevertheless, while antigen uptake is a point in favor, it is by no means a guarantee of an immune response, as the gut environment is highly tolerogenic. This is one of the main challenges in oral vaccine development, which we will need to face further down the pipeline (10, 34).

At this stage, the other issue studied here regarding the potential use of NPs was whether they could evoke an antiviral innate immune response. We therefore stimulated ZFL cells with the three viral antigen NPs and the control iRFP^NP^ and checked expression of innate immune gene markers of viral infection: IFN-stimulated genes (35) including transcription factors *irf7* and *stat1b* and genes encoding antiviral peptides *mx* and *viperin* (*vig1*) (36), as well as *gig2* and chemokine *ccl4*. The viral dsRNA mimic, poly(I:C), was used as a positive control as it mounts an antiviral response in zebrafish (29) among other species, and as such is being tested as a potential fish vaccine adjuvant (37). The results for VNNV-C^NP^ were particularly promising. All six genes tested were highly upregulated, attaining similar levels to those obtained with poly(I:C). IPNV-VP2^NP^ also caused significant but much lower up regulation, while VHSV-G-frg16^NP^ only upregulated three of the genes at lower levels. Upregulation by the control NP, iRFP^NP^ was slight or negligible. The poly(I:C) positive control was not conceived for direct quantitative comparison, as it mimics nucleic acid, not protein. For this reason, we were surprised that the upregulation of the innate immune genes tested appeared so similar, between VNNV-C^NP^ and poly(I:C). Multiple activation pathways are triggered by viral infection (38), but we had not expected such a comparable profile of gene upregulation by the recombinant protein and the viral dsRNA mimic. Apparently, we had achieved an innate antiviral response in full swing, by two quite different stimuli.

Indeed, the role of viral capsid proteins in innate immune stimulus is starting to be elucidated by research in mammalian systems. It appears that innate immune activation can be mediated by recognizing the intrinsic order of capsid structure. For instance, TRIM5 has been reported as a pattern recognition receptor, specific for retrovirus capsid lattice (39). Furthermore, toll-like receptor 2 has recently been shown to respond to the multi-subunit arrangement of viral capsids, independent of amino acid sequence, or specific morphology. Rather, stimulus relies on repeating protein subunits, as a conserved common denominator across viral capsids (40). We do not know how well our NPs fit into this descriptor, but IBs are entities composed of repeated subunits in an ordered nanostructure. Fourier transform infrared microspectroscopy shows that IBs are proteins with native-like structure entrapped in densely packed intermolecular β-sheet bridges (41). The relative amount of native-like protein can differ with production conditions. Out of interest, we checked crystallography data from a VLP of Grouper nervous necrosis virus (42), another marine betanodavirus. The self-assembled particle size is typical of the *Nodaviridae* 30–35 nm, and the shell domain has the common viral capsid protein jelly roll structure with eight β strands forming two antiparallel sheets (43). Our VNNV-C^NP^ is considerably larger (~420 nm) than the VLP and we do not know the 3D structure further than the order inferred from the FESEM images. We also do not know if there is self-assembly of the native-like viral capsid protein as it emerges from the IB scaffold. Nevertheless, our results imply that this NP triggered an innate immune response in ZFL cells as if it were a virus.

It should also be pointed out that the NPs, while made mainly of viral protein subunits, contain low amounts of bacterial nucleic acids, peptidoglycan, and lipopolysaccharide (14). The non-relevant immune control, iRFP^NP^ also has these contaminants but was a poor stimulator of the antiviral response both in ZFL and HKM cells. This does not preclude stimulus of other genes. In fact, in prior work, when iRFP^NP^ was injected in zebrafish and a challenge with *P. aeruginosa* was performed, there was significant survival of treated fish compared with control. The protection was presumably due to stimulus evoked by these contaminants (14).

Regarding IPNV-VP2^NP^ and VHSV-G-frg16^NP^, the important consideration for our purposes was that the NPs could stimulate the chosen viral response gene markers, more than the size of the effect. In this vein, we were concerned that VHSV-G-frg16^NP^ had not produced stimulus in several of the genes tested in ZFL. It should be kept in mind that this NP construct is not the whole antigenic protein, in contrast to the other NPs, but it has antigenic epitopes including Mx inducing sites (18). Given that tropism might be a significant factor, we tested the expression of viral response gene markers, induced by IPNV-VP2^NP^ and VHSV-G-frg16^NP^ in RT-HKM primary cultures, as trout is a natural host for IPNV and VHSV. In these experiments, we included *ifit5* (27) and *mda5* (35) an IFN-induced gene and a dsRNA receptor belonging to the RIG-1-like receptor family, respectively. In this case, we got significant stimulus of all the gene markers, at a similar level for both NPs.

Summarizing, we have produced three recombinant viral antigenic proteins as nanostructured biomaterials with view to use in orally delivered prophylaxis. The methodology employed is straightforward, cheap, and fully scalable. These “nanopellets” are successfully taken up *in vitro* in ZFL and *in vivo* in zebrafish *via* oral administration. They stimulate an antiviral innate immune response both in ZFL and RT-HKM cells. They therefore are candidates for immunostimulants. On the road to vaccine development, the next essential steps are to run protection studies and to demonstrate the raising of antigen-specific antibodies in target fish species. We are keen to further explore their potential.

## Ethics Statement

All experimental procedures were approved by the Human and Animal Experimentation Ethics Committee of the Universitat Autònoma de Barcelona (Reference 1533) and were done in strict accordance with the recommendations of the European Directive (2010/63/EU) on the protection of animals used for scientific purposes.

## Author Contributions

RT, DT, and JJ performed the experiments. RT and JC designed the constructs. JJ designed the zebrafish intubation method. NR, RT, and DT designed the other experiments and RT did the data analysis. RT and NR wrote the paper. All the authors were involved in discussions and contributed to the writing of the final manuscript.

## Conflict of Interest Statement

The authors declare that the research was conducted in the absence of any commercial or financial relationships that could be construed as a potential conflict of interest.
