# Supplementary material for: Protein Nanoparticles Made of Recombinant Viral Antigens: A Promising Biomaterial for Oral Delivery of Fish Prophylactics
Source: Front Immunol. 2018 Jul 18;9:1652. doi: 10.3389/fimmu.2018.01652 (PMC6060434; doi:10.3389/fimmu.2018.01652)
Supplement: Supplementary file 1 [file Data_Sheet_1.doc]

Supplementary Material

**Protein nanoparticles made of recombinant viral antigens: a promising biomaterial for oral delivery of fish prophylatics**

*Rosemary Thwaite, Jie Ji, Debora Torrealba, Julio Coll, Manel Sabès, Antonio Villaverde and Nerea Roher^*^*

*** Correspondence:** Corresponding Author: nerea.roher@uab.es

# Supplementary Figures and Tables

**Figure S1: Efficiency of fluorescent labeling of NPs**

**Figure S2: Western blots of NP protein production time course**

**Figure S3: MTT assay of nanopellet (NP) cytotoxicity in ZFL**

**Table S1: Primers used for real time PCR**

**Figure S1**

**
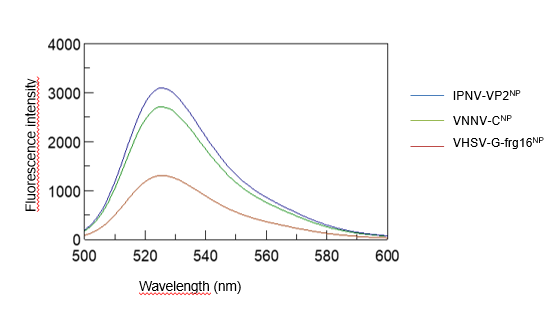
**

**Figure S1. Efficiency of fluorescent labeling of NPs:** Post labeling with Atto-488 NHS ester, each NP (100 µg/ml) was treated O/N with 6M guanidinuim chloride at RT and fluorescence intensity was read on a Jasco FP8200 fluorometer.

**Figure S2**

**
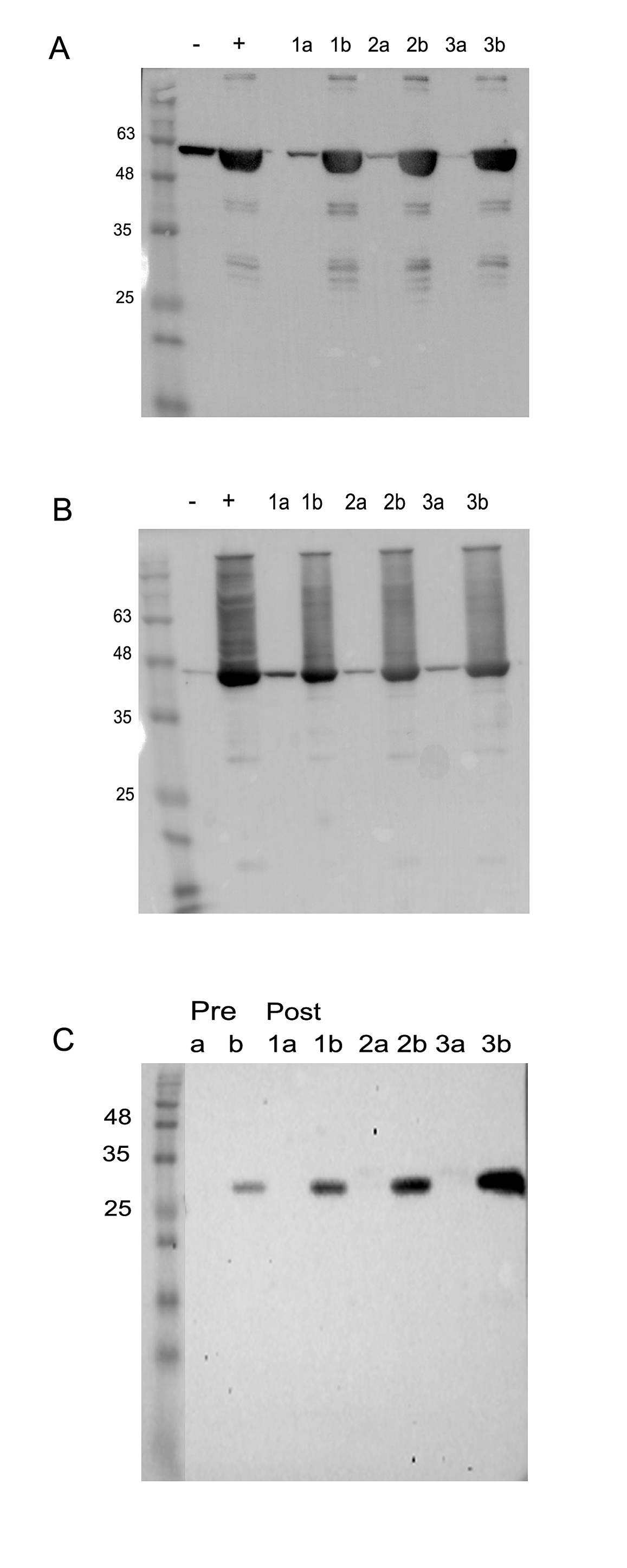
**

**Figure S2:** **Western blots of NP protein production time course:** Cultures were set up as described (section 2.1.2) and induced with 1 mM IPTG. Samples taken pre-induction and at every hour post induction for 3 h. Cells were collected by centrifugation, resuspended in PBS plus protease inhibitor (cOmplete Tablets, Roche) and sonicated (total protein production). To obtain the soluble and insoluble fraction equivalent amounts of cells (volume of cell culture adjusted to the same OD for every time point in A and B) were centrifuged, resuspended in PBS and then sonicated. Sonicated cells were centrifuged 14,000 g at 4ºC for 15 min. Supernatant was retrieved as the soluble fraction, and the pellet, (insoluble fraction) was resuspended in the same volume of PBS. Equal aliquots were run on a western and NPs detected using an anti-His-tag antibody.

**A** **and B:** Production of IPNV-VP2^NP^ and VNNV-C^NP^ respectively. Total protein production pre-induction (-) and 3 h post IPTG induction (+) for the same volume of culture sample. Soluble (a) and insoluble (b) fractions at 1, 2 and 3 h post IPTG induction for the equivalent amount of cells at all time points.

**C:** Production of VHSV-G-frg16^NP^. Soluble (a) and insoluble (b) fractions, pre-IPTG induction and 1, 2 and 3 h post IPTG induction for the same volume of culture at all time points.

The amount of protein increases sharply with IPTG induction and protein is almost entirely in the insoluble fraction (inclusion bodies). Predicted molecular weight of each protein using Expasy Protparam is 49.6, 38.2 and 30.4 kDa, for A, B, C.

**Figure S2 cont.**

**D.** Original western used for figure S2 C. The ladder was cut and pasted to be next to the tracks of interest showing production of VHSV-G-frg16^NP^ (30.4 kDa). Other tracks (1-6) are not this protein, nor the same type of experiment. Westerns A and B in S2 are originals.


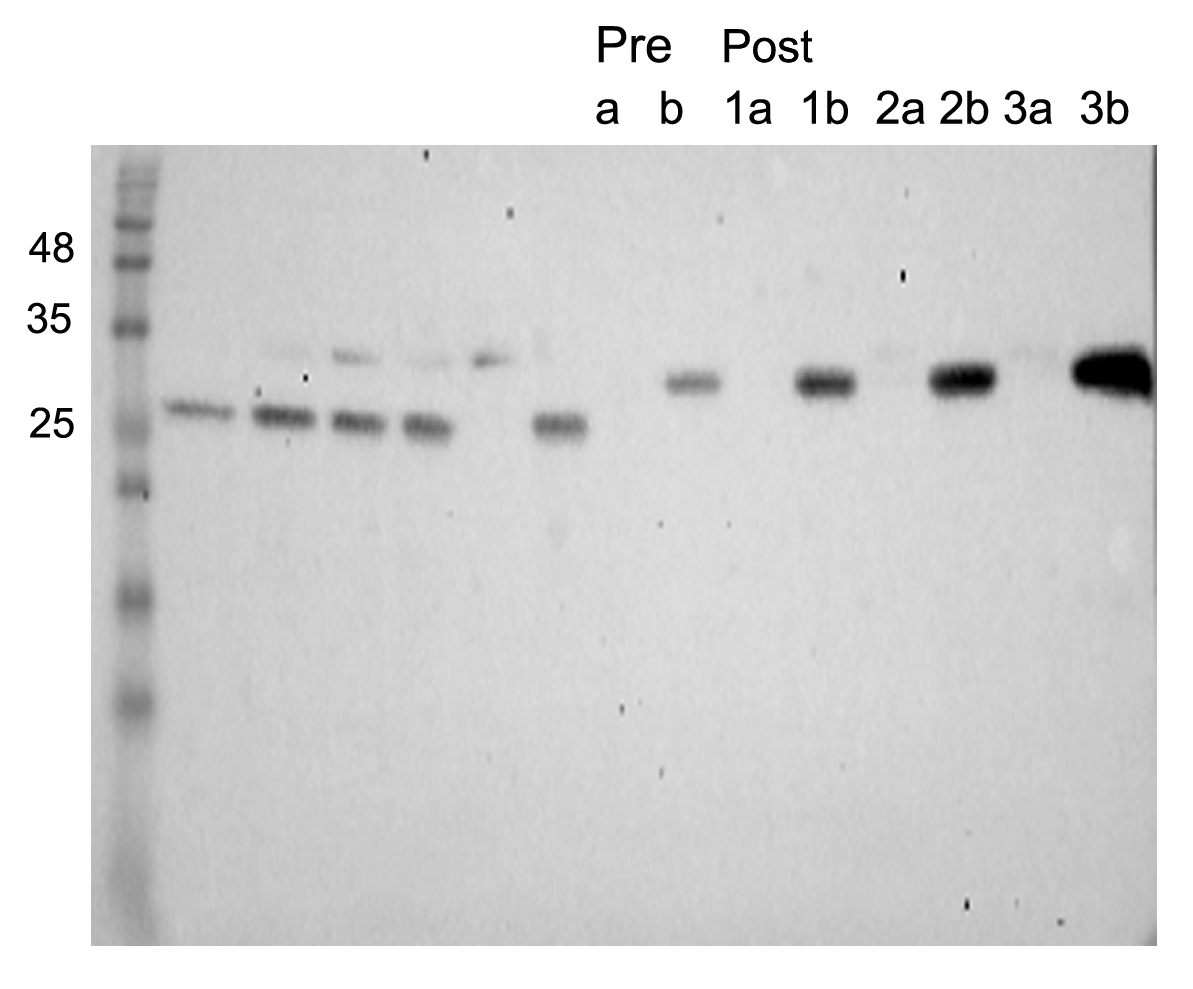


**Figure S3**

**
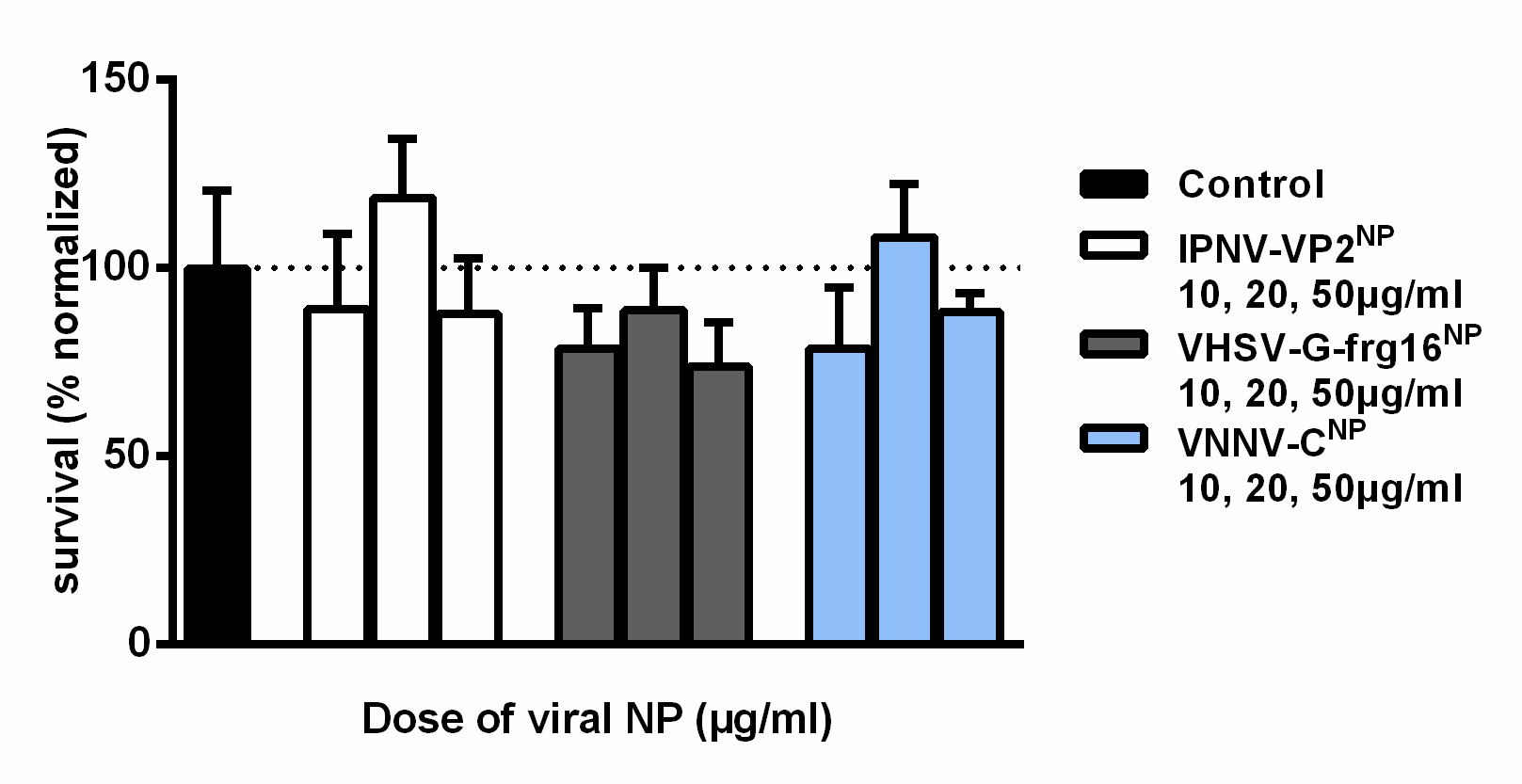
**

**Figure S3. MTT assay of nanopellet (NP) cytotoxicity in ZFL:** Zebrafish liver (ZFL) cells were stimulated with viral NPs at 10, 20 and 50 µg/ml in triplicate for 14 h at 28 ºC. Positive control was no NP added. Negative control was cells treated with 1 % Triton. After MTT treatment, 6h incubation and then solubilization in DMSO, absorbance was measured at 550 nm and data normalized with positive and negative control readings set to 100 % and 0 % viability respectively. The experiment was repeated twice. A one-way ANOVA was performed with Dunnett’s multiple comparisons test between each treatment and control mean, at significance level p<0.05. No treatment group was significantly different from control.

**Table S1 Primers used for real time PCR**

| **Zebrafish primers** |  |  |  |
| --- | --- | --- | --- |
| **Gene** | **Sequence (5´-3´)** | **Product size (bp)** | **Accession # / reference** |
| *ef1-α* | FW_CTTCTCAGGCTGACTGTGC | 133 | AY422992 |
|  | RV_ACGATCAGCTGTTTCACTCCC |  |  |
| *mx* | FW_ACATCTTGGATCGTTCAGGGGA | 163 | NM_182942.4 |
|  | RV_AACGCAGGTTCCTCCAACAG |  |  |
| *viperin* (*vig1/ rsad2*) | FW_CTTATAGGTCGAGCACAGGGC | 165 | NM_001025556.1 |
|  | RV_ACGTACTGGATTGAGAGCGGTG |  |  |
| *gig2* | FW_AGGGTACGACACTGCCTGGT | 148 | NM_001245989.1 |
|  | RV_ AGGGTCACCAAAGCCACAAT |  |  |
| *irf7* | FW_GAGCAAATACGCTTCCCGA | 141 | NM­_200677.2 |
|  | RV_CTTGTCCTGACGAAAGCCATA |  |  |
| *stat1b* | FW_TCCCAATGGAGATCCGACAAT | 107 | NM_200091.2 |
|  | RV_CAGGAGCTCATGGAAGCGAAC |  |  |
| *ccl4* | FW_CATGACAAGCCAGCAGTGCC | 126 | NM_001129894.1 |
|  | RV_ACACGTTTGCTGTCAATGGCCTG |  |  |
| **Rainbow trout primers** |  |  |  |
| *ef1-α* | FW_CAAGGATATCCGTCGTGGCA | 327 | NM_001124339.1 |
|  | RV_ACAGCGAAACGACCAAGAGG |  | [1] |
| *mx* | FW_ATGCCACCCTACAGGAGATGAT | 127 | NM_001171901.1 |
|  | RV_TGCAGCTGGGAAGCAAACTCC |  |  |
| *vig1* | FW_AACGCTGGGGAGAACAGTCT | 181 | NM_001124253.1 |
|  | RV_TCCCCTCTCGGCAATCCA |  |  |
| *ifit5* | FW_GGGTAGCCTATTCCGCGTACTT | 80 | NM_001124333 |
|  | RV_CTGCTTTGACCGAGGCACTC |  |  |
| *mda5* | FW_ TTTGTGCTGAGCATCTACGG | 148 | NM 001195179.1 |
|  | RV_TTAATGATGGCCTCCTCGTC |  |  |
| *ccl4* | FW_TGTTCACCCCTCGTCTTGCT | 104 | NM_001124489.2 |
|  | RV**_** ACATTTCTTCGGTCCGCTTG |  |  |

[1] Tacchi, L., Bickerdike, R., Secombes, C.J., Pooley, N.J., Urquhart, K.L., Collet, B. & Martin, S.A.M. 2010 Ubiquitin E3 ligase atrogin-1 (Fbox-32) in Atlantic salmon (Salmo salar): Sequence analysis, genomic structure and modulation of expression. *Comparative Biochemistry and Physiology, Part B* **157**, 364-337. (doi:10.1016/j.cbpb.2010.08.004).
